# Regulation of phosphorus bioavailability by iron nanoparticles in a monomictic lake

**DOI:** 10.1038/s41598-018-36103-x

**Published:** 2018-12-10

**Authors:** H. Saeed, A. Hartland, N. J. Lehto, M. Baalousha, M. Sikder, D. Sandwell, M. Mucalo, D. P. Hamilton

**Affiliations:** 1 Environmental Research institute, School of Science, Faculty of Science and Engineering, University of Waikato, Hamilton, Waikato New Zealand; 2 Department of Soil and Physical Sciences, Faculty of Agriculture and Life Sciences, Lincoln University, Lincoln, New Zealand; 3 Center for Environmental Nanoscience and Risk, Department of Environmental Health Sciences, University of South Carolina, Columbia, SC United States; 4 Chemistry, Faculty of Science and Engineering, University of Waikato, Hamilton, Waikato New Zealand; 5 Australian Rivers Institute, Griffith University, Griffith, Australia

## Abstract

Dissolved reactive phosphorous (DRP) in lake systems is conventionally considered to predominate over other dissolved P species, however, this view neglects an important set of interactions that occurs between P and reactive iron hydroxide surfaces. This study addresses the coupling of P with dispersed iron nanoparticles in lakes, an interaction that may fundamentally alter the bioavailability of P to phytoplankton. We used diffusive gradients in thin films (DGT) and ultrafiltration to study Fe-P coupling in the water column of a monomictic lake over a hydrological year. Fe and P were predominantly colloidal (particle diameters > ~5 nm < ~20 nm) in both oxic epilimnetic and anaerobic hypolimnetic waters, but they were both DGT-labile under sub-oxic conditions, consistent with diffusion and dissolution of Fe-and-P-bearing colloids within the DGT diffusive gel. During peak stratification, increases in Fe and P bioavailability were spatially and temporally coincident with Fe nanoparticle dissolution and the formation of a deep chlorophyll maximum at 5–8 m depth. These results provide a window into the coupling and decoupling of P with mobile iron colloids, with implications for our understanding of the behaviour of nutrients and their influence on phytoplankton community dynamics.

## Introduction

Phosphorus (P) and nitrogen (N) play a central role in the biological productivity of aquatic systems^1–3^. In freshwaters, dissolved inorganic N and P act as limiting nutrients depending on their molar ratios^4^, but it is usually P that limits the productivity of terrestrial aquatic systems.

Excess P availability occurs primarily through human actions^2,5^, with the widespread use of P fertiliser, poor riparian buffering and uncontrolled sedimentation being major drivers^5,6^. Despite regulation of anthropogenic P inputs, the biogeochemical cycling of P between lake water columns and sediments typically maintains high P availability to phytoplankton^7^.

Phosphorus is present in a range of inorgaimnic and organic species across the dissolved, colloidal and particulate size ranges^8^. Organic P constitutes a major part of the dissolved P pool^9^, while particulate (>1 μm) and colloidal (1–1000 nm) fractions (from plant, animal and bacterial sources) can be present as poly-phosphates adsorbed to metal oxides, hydroxides and clays^10–12^.

Bioavailable P is usually the sum of the P species that are immediately available for biological uptake, or species that are easily transformed into available form(s) by naturally occurring processes^13–15^. Thus, the bioavailable P fraction is a moving target subject to biological, chemical and physical processes that vary through time in lakes and which requires cautious monitoring to be meaningfully estimated.

During lake stratification events, organic matter (OM) is supplied to the hypolimnion through settling of suspended particulates, or bioturbation in the upper few mm of the sediment^13,16^ or turbulent mixing across the thermocline^17,18^. To-date, two distinct phenomena have been proposed to explain the observed P fluxes from benthic sediments, (1) elevated pH (resulting from algal photosynthesis) favours the desorption of PO_4_^3−^ from sediment solids^19,20^ and (2) reductive dissolution of Iron(III) species at the onset of anaerobic conditions^17^. The mineralisation of OM consumes oxygen and creates steep redox gradients between iron species present in sediments and water column. These gradients are expected to affect the distribution and bioavailability of P because of well-documented interactions between dissolved inorganic phosphorus (DIP) and Fe hydroxide surfaces^21,22^.

Iron oxide colloids often form by hydrolysis and oxxidation of Fe(II) at oxic/anoxic boundaries in lakes^17,23^. Differences in the surface properties of ferrous (Fe(OH) _2_) and ferric (Fe(OH) _3_) colloids are likely to be significant for P binding^24^. The size, structure, and reactivity of this hydrolysis product can vary, e.g., because of natural organic matter coatings^25,26^. Previous studies of other freshwater ecosystems have reported mobile Fe colloids to be in the size range of 0.05 to 0.5 μm^27–31^. Such Fe colloids have been shown to be stable in sub-oxic conditions^32,33^ and can therefore potentially survive for extended periods under anaerobic conditions, with concomitant effects on solution chemistry. Hence, aquatic colloids can be rich in both P-binding surfaces and organic matter (the latter containing a significant amount of organic P)^34^.

**Figure 1:**
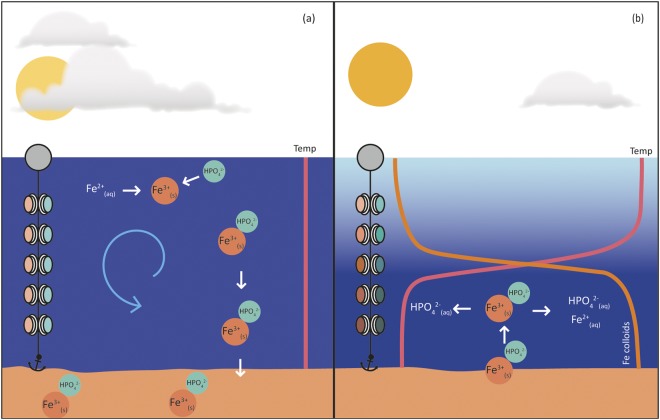
Conceptual figure summarising the biogeochemical cycling of iron (Fe) and phosphorus (P) in Lake Ngapouri, with DGT probes arrayed through the water column to detect changes in labile Fe and P concentration. In (**a**) oxidation of Fe^2+^ and co-precipitation of Fe^3+^ and P (and/or P adsorption on Fe hydroxide colloids) occurs during isothermal winter conditions, leading to Fe and P sedimentation and uniformly low dissolved Fe and P concentrations through the water column. In (**b**) the release of Fe colloids in the deep hypolimnion during summer stratified conditions is depicted (right and left arrows show products of reductive dissolution of Fe colloids and P desorption, respectively).

Distinct from gravitoids (large particles >10 μm^27^, colloids (1–1000 nm) and nanoparticles (1–100 nm) remain suspended for extended periods and thus provide surface sites for adsorption or other chemical reactions within the water column^10^. When present within the lake water column, these dispersed surfaces have the potential to significantly modify P bioavailability to phytoplankton (Fig. 1). While the coupled release of Fe and P has been previously demonstrated in lake sediments^35,36^ the significance of Fe colloids for P bioavailability has yet to be elucidated in these systems.

The dearth of studies on the interaction between P and colloids in lakes can be explained by inherent difficulties associated with probing colloidal interactions in such sub-oxic (reduced) domains. This study seeks to address the lack of information on colloidal Fe-P interactions under reduced conditions, and the absence of highly spatially- and temporally-resolved data on P speciation in lake systems in general, by combining sensitive and representative measures of *in-situ* water column chemistry based on diffusive gradients in thin films (DGT) and ultrafiltration methods.

The use of diffusive gradients in thin films (DGT) has gained recognition as an alternative approach for rapid evaluation of labile metal/nutrient content in water and sediments. DGT solution probes are small plastic devices containing a filter membrane, ion permeable hydrogel and a binding agent incorporated in a basal gel layer. When immersed in solution, the solutes pass through the hydrogel of known thickness (*Δ*g), also referred as diffusive layer/gel, and accumulate at the binding gel layer. Dissolved species concentrations are then determined by using Fick’s first law of diffusion from measured mass (M) over deployment time (t)^37^. These devices formed the principle means by which the biogeochemical dynamics of Fe and P in Lake Ngapouri were deduced. For further information on the use of DGT for evaluating bioavailability we refer readers to Zhang & Davison^38,39^ and Zhang *et al*.^40^.

## Results and Discussion

### Physicochemical characteristics of Lake Ngapouri

**Figure 2:**
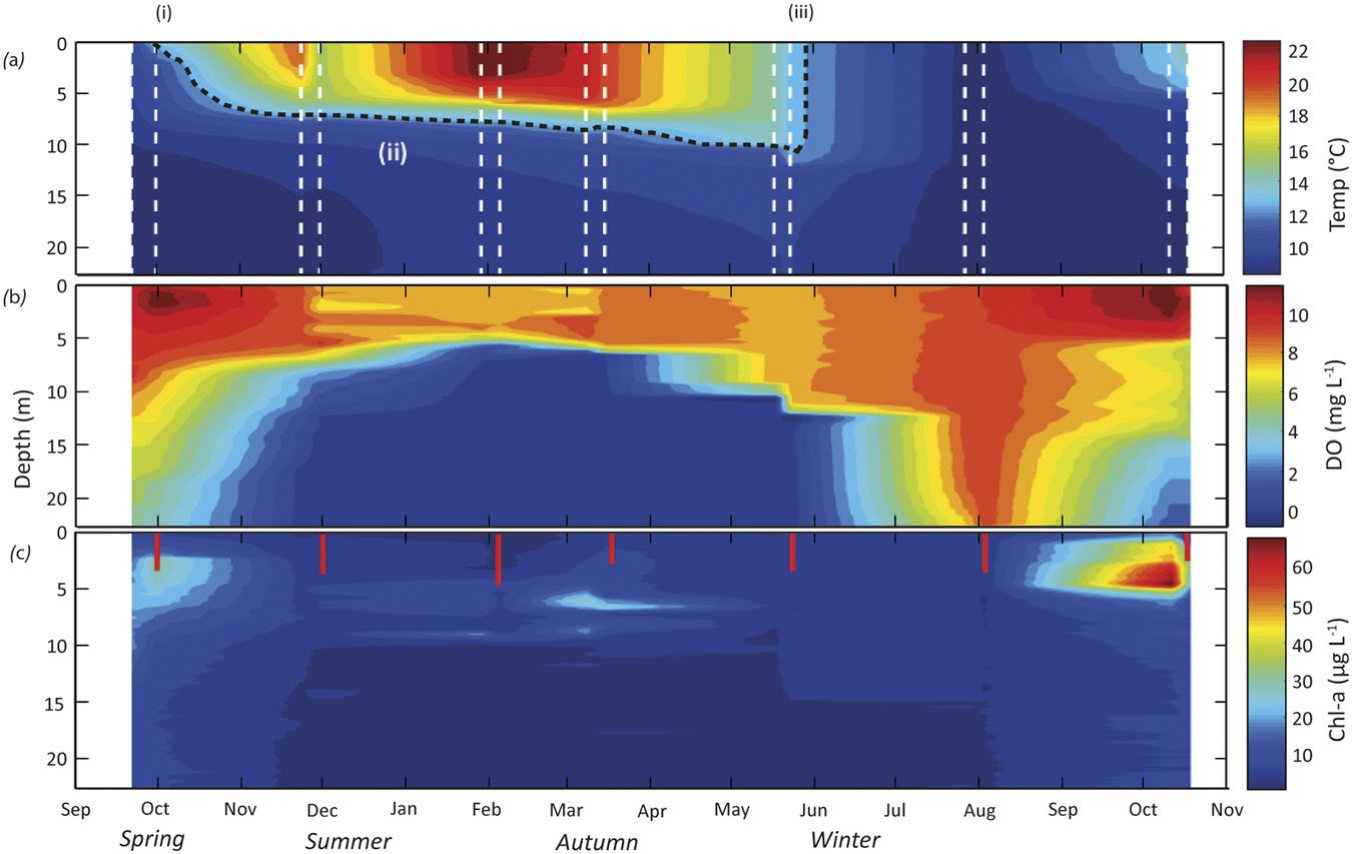
Physicochemical characteristics of the water column of Lake Ngapouri between September 2015 and October 2016. (**a**) Temperature, (**b**) dissolved oxygen (DO), and (**c**) chlorophyll fluorescence data were extracted from CTD casts conducted before and after each DGT deployment. Data points are interpolated using the MATLAB contour function. Vertical lines in (**a**) (broken white) and (**c**) (solid red) show the time of sampling and Secchi depth respectively. The broken black line in (**a**) shows the timing of the onset (i) and end (iii) of stratified conditions in the lake as well as the approximate position of the thermocline (ii) to aid visual identification of these features.

Stratification in Lake Ngapouri began in November 2015 (Fig. 2) and the lake water became markedly clearer at this point compared with the preceding winter mixed period. High turbidity during the winter may have been caused by the resuspension of sediment or a predominance of diatomaceous algae (Fig. 2), whereas increased clarity during summer can be plausibly explained by coagulation and sedimentation of the suspended particles^41^.

The surface water temperature of Lake Ngapouri ranged between 9 °C and 23 °C over the study period. The annual temperature cycle of the lake was characterised by two distinct phases: isothermal conditions and thermal stratification (Fig. 2).

CTD (Conductivity, Temperature, Depth profiler) casts indicate that isothermal conditions continued until September, and that a thermocline started to establish at the start of November 2015. Thermal stratification established as the summer progressed (Fig. 2).

The exact depth of the CTD casts varied due to changes in water levels over the course of this study. Therefore, the time series presented here was reduced to the minimum depth recorded in the sampling period (22 m from surface) for consistency. Since profiles for chlorophyll fluorescence were determined by CTD casts and no CTD data were collected at night time we were not able to determine the direct effect of non-photochemical quenching^42^. Chlorophyll fluorescence data indicate periods of high phytoplankton biomass during the winter mixed period in September 2015 and October 2016, and a peak just above the thermocline (ca. 5–8 m) during March-May 2015. This deep chlorophyll maximum (DCM) was quite similar to that observed in nearby Lake Okaro, a lake of similar trophic status with an anaerobic hypolimnion^43^. No response in chlorophyll fluorescence was observed after the diurnal mixing event in November 2015.

Once established, the thermocline was very stable and progressively deepened from 5 to 13 m from November 2015 to March 2016, respectively. The DO concentration markedly decreased below 5 m depth in November 2015 and established a completely anaerobic hypolimnion extending from 15 m depth to the bottom of the lake over the same interval. The epilimnion deepened to about 10 m by May 2016 and the lake experienced complete seasonal mixing in July 2016.

The lake water pH was almost uniform during winter mixed conditions, but started to decrease in deeper waters of the lake during early stratification. The onset of stratification coincided with elevated chlorophyll fluorescence near the lake surface, which coincided with higher pH conditions (exceeding pH 9 in October 2016; Fig. S2, supplementary information).

### Colloidal size distributions, morphology and chemistry through time

#### AFM Results

**Figure 3:**
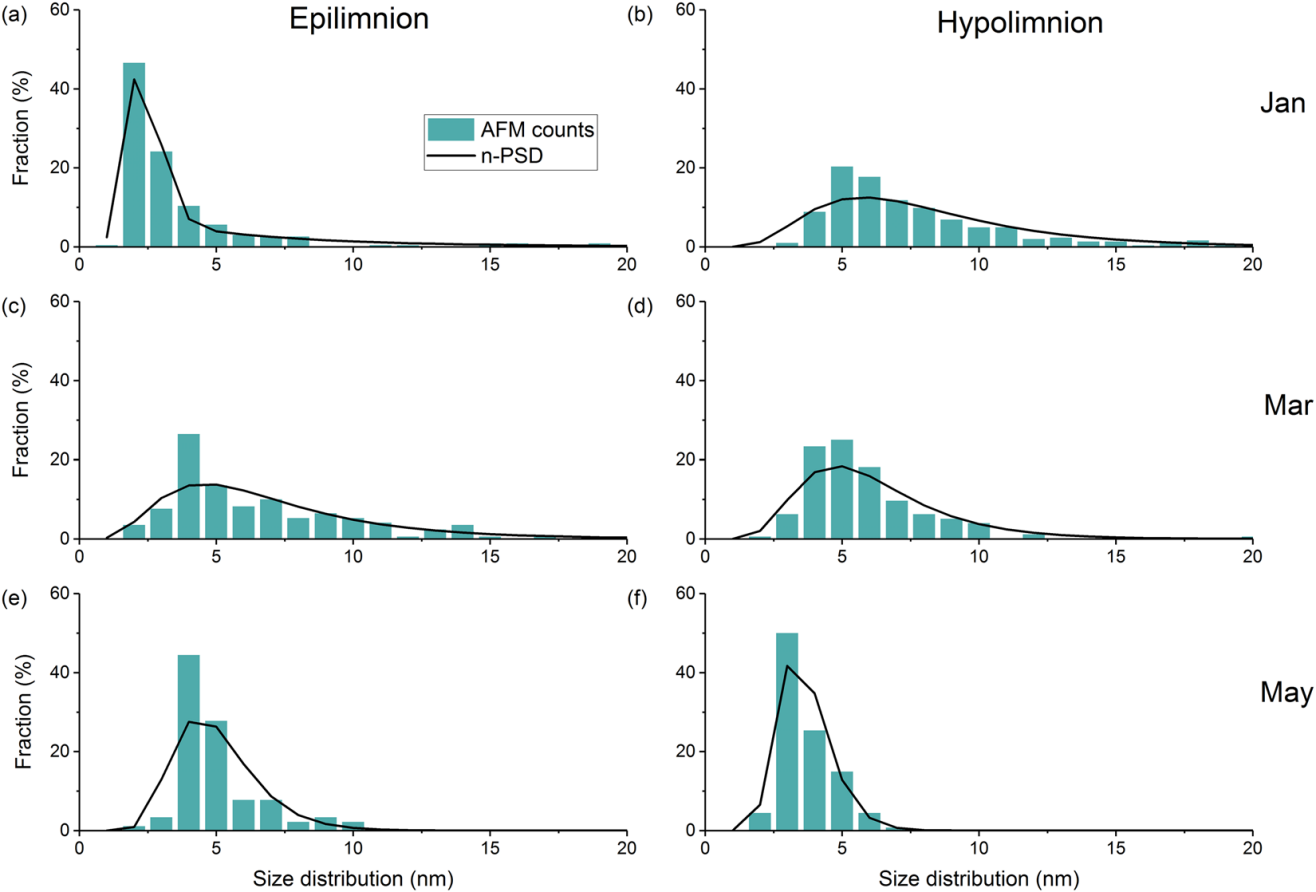
Atomic force microscopy (AFM) particle size distributions (PSD) from Lake Ngapouri samples collected between January and May 2016. First column are epilimnetic samples from (**a**) January, (**c**) March, (**e**) May and in the second column hypolimnetic samples from (**b**) January, (**d**) March, and (**f**) May 2016, respectively. Fraction (%) gives the percentage of total particles detected by AFM image analysis.

Particle size distributions (PSD) measured by atomic force microscopy (AFM) of colloids are shown in Fig. 3. It should be noted that AFM is biased toward small particles and does not necessarily cover the entire colloid size distribution range. Colloids were predominantly in the size range of 1 to 10 nm in both epilimnetic and hypolimnetic samples. In the epilimnion during January 2016, particles in the 2–3 nm size range constituted >45% of total particle numbers. Particles >10 nm did not form a major proportion of the PSD during this month (Fig. 3) which may be related to particle settling and gravitoids sedimentation under the prevailing stratification. On the other hand, in the hypolimnion during the same month colloid sizes shifted toward larger particle sizes (between 3 and 18 nm) with particles between 4–6 nm constituting the most significant fraction at ~40% of all measured particles (Fig. 3).

In the epilimnion during March 2016, particles were in the size range of 2–14 nm, with the greatest density between 4–5 nm (Fig. 3c). In the hypolimnion, particles remained distributed within a narrow size range, with those in the 4–7 nm range making up ~50% of the total particle number (Fig. 3e). In May, the epilimnion PSD centred on 3–9 nm, with these particles constituting more than 50% of the total particle number (Fig. 3a). This PSD was comparable to the hypolimnion PSD in January and March. The consistent size distributions between months in the hypolimnion might be explained by: (1) resuspension of the particles from the bed sediments during this stagnant phase, or (2) the recycling of nanoparticles by cycles of reduction and precipitation within the water column^44^. Generally, a trend was observed toward decreased particle size in the deeper anaerobic water during stratification. This could be consistent with the reductive dissolution of larger colloids and precipitation of fresh nano-scale Fe (II)/Fe (III) hydroxides, although this cannot be substantiated without higher spatial and temporal resolution data.

#### TEM Results

**Figure 4:**
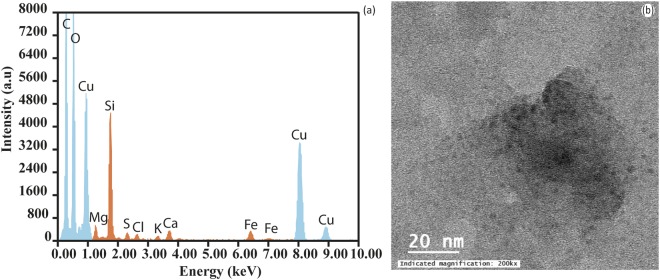
Representative TEM results from Lake Ngapouri showing abundant nano-scale colloids containing iron and silicon. (**a**) The accompanying EDX spectrum from the globular colloid photomicrograph shown in (**b**).

The Transmission Electron Microscope (TEM) analysis confirmed that Fe-bearing particles were the most prevalent class of nanoparticles detected in Lake Ngapouri. Figure 4 shows a representative TEM micrograph and EDX (Energy-dispersive x-ray analysis) spectrum, which show peaks for Fe, O with contributions from Si; all major elements commonly associated with colloidal species in freshwaters. Evidence was also found for the presence of Fe monosulfides in the hypolimnion as would be expected at the interface of Fe-reducing and S-reducing zones in the water column and sediments.

### Iron depth distributions through time

Water sample aliquots collected after ultrafiltration in a zero-grade N_2_ atmosphere were used to measure the fraction of Fe and P associated with colloidal particles. This analysis provides an approximation of the concentration of Fe and P in the colloidal range which excludes particles >0.45 μm and nanoparticles <~5 nm diameter (based on 100 KDa ultrafiltration).

**Figure 5:**
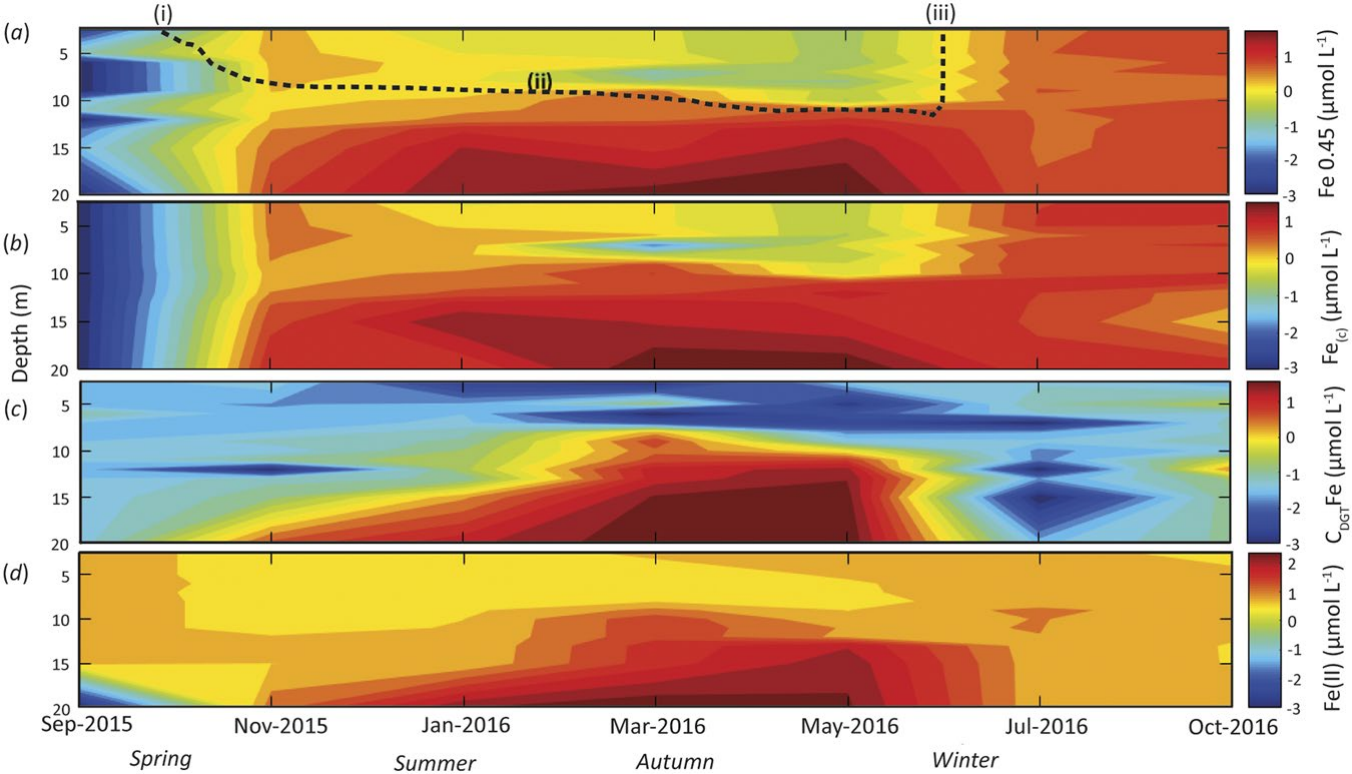
Time series of the depth-distribution of iron (Fe) at log_10_ scale in operationally-defined water fractions from Lake Ngapouri, (**a**) Dissolved Fe (μmol L^−1^), (**b**) Colloidal Fe _(c)_ (μmol L^−1^), (**c**) C_DGT_ Fe (μmol L^−1^) and (**d**) Fe (II) by Ferrozine method (μmol L^−1^). Broken black line in (**a**) shows the timing of the onset (i) and end (iii) of stratified conditions in the lake as well as the approximate position of the thermocline (ii).

Time series of Fe depth distributions in Lake Ngapouri (Fig. 5) reveal the dominant influence of stratification on Fe cycling. The Fe concentration started to build up in all fractions (dissolved, colloidal, C_DGT_ and Ferrozine) over the period of early stratification and was maintained during summer. Fe < 0.45 μm was predominantly colloidal (Fig. 5b). Taken together with the AFM and TEM results, this implies that Fe was present in the hypolimnion either as simple inorganic colloids, e.g. Fe(III) hydroxides with particle sizes typically ≥5 nm, or in association with macromolecular organic colloids/aggregates. Analysis of the organic fraction of the dissolved and colloidal size range in the total organic carbon (TOC) measurement returned colloidal TOC concentrations in the 0.1–3.31 ppm range, constituting up to 100% of the organic carbon <0.45 mm (i.e., dissolved organic carbon (DOC)) in some cases.

DGT is thought to measure (1) all hydrated free-ion species, (2) a vast majority of the colloidal and complexed species that may be released into free-ion species during the deployment and (3) colloidal species that can diffuse through the APA hydrogel and bind with the basal resin layer^38,45^. The diffusion coefficient of solutes in the hydrogel decreases with an increase in their hydrated radii. Although exact information on the porosity of APA diffusive gel is lacking, initial studies by Zhang and Davison^37^ reported the pore size to be >5 nm while Fatin-Rouge, *et al*.^46^ gave indicative pore sizes in the 50–74 nm range, depending on the gel cooling rate during polymerization. Furthermore, particles >60 nm were considered locally trapped and not evenly diffused through the gel^46^. However, Davison and Zhang^38^ suggested that despite the probable existence of larger pore sizes, DGT devices can only measure particles <2 nm size because of the slow diffusion rate of larger particles. In addition, the structure and composition of solutes are important determinants. Thus, DGT can be considered to be sensitive to nano-scale particles which are sufficiently labile to be measured by the DGT resin layer, or otherwise become embedded in the resin gel matrix^39^. The diffusion coefficients (D) of nanoparticles in the size range determined by AFM were calculated using Stokes law (Table 1). These *D* values were used to calculate the time to reach 95% of the steady state accumulation rate (T_95%_) for colloids. *D* values from Zhang and Davison^37^ were used to calculate T_95%_ for dissolved Fe (Table 2). The T_95%_ values are comparable for the colloids >1 nm and demonstrate that over the course of a week (typical duration of deployment in this study), DGT is likely to have accurately recorded the concentration of DGT-labile colloidal species in the lake.

**Table 1:** Calculation of the diffusion coefficient (D) of the nanoparticles in the size range determined by AFM assuming spherical morphology.

Month	Compartment	Temp.(K)	Average particle radius (nm)	1σ	Đ	Average colloid	Solute
						D (×10^−6^ cm^2^ s^−1^)	D (×10^−6^ cm^2^ s^−1^)
January	Epilimnion	295.15	0.61	3.47	2.86	4	5.63
March	Epilimnion	283.15	4.49	4.25	0.47	0.52	3.19
May	Epilimnion	294.15	6.45	5.43	0.41	0.37	5.47
January	Hypolimnion	283.15	4.8	4.93	0.51	0.49	3.19
March	Hypolimnion	287.15	2.25	1.53	0.34	1.05	4.45
May	Hypolimnion	283.15	1.58	0.98	0.31	1.47	3.19

1σ = 1 standard deviation (*n* = no. measurements); Đ = dispersity.

**Table 2:** Comparison of the time taken by DGT to reach 95% steady state accumulation rate for Fe using representative diffusion coefficients for colloids or solutes (i.e. hydrated Fe^2+^ ions) in the epilimnion and hypolimnion of Lake Ngapouri.

Epilimnion	Colloid	T_95%_ (hrs)	Hypolimnion		T_95%_ (hrs)
Solute			Solute	Colloid	
T_95%_ (hrs)	particle radius (nm)		T_95%_ (hrs)	particle radius (nm)	
18	0.61	26	21	0.61	30
32	4.49	198	37	4.49	227
19	6.45	278	22	6.45	319
32	4.8	211	37	4.8	242
23	2.25	98	27	2.25	112
32	1.58	70	37	1.58	80

T_95%_ (hrs) = time taken by DGT to reach 95% steady state concentration.

Direct measures of the initial increase in Fe concentration in the hypolimnion of Lake Ngapouri between November and January (Fig. 5) were not mirrored by an increase in C_DGT_ Fe, which appeared to lag colloidal Fe in the upper hypolimnion (Fig. 5c). Because DGT only measures the diffusible Fe concentration, it follows that Fe released into the hypolimnion in the early period of stratification was non-DGT-labile. This supports the interpretation that Fe became more labile through time in the hypolimnion as reducing conditions intensified (Figs 2 and 5). Following March 2016, Fe concentrations measured by DGT showed improved agreement with the other Fe fractions. Indeed, in the latter months of stratification it appears that although Fe was predominantly colloidal (Fig. 5b), it was also DGT labile (Fig. 5c).

It is clear from Fig. 5 that the increase in Fe (II) started from the bottom of the lake, consistent with diffusion from the lake sediment/benthic nepheloid layer, which was characterised by lower DO concentrations than the overlying hypolimnetic water in the early period of stratification^30^. Dissolved Fe (II) first appeared in the lowermost sample in late November 2016 (Fig. 5), when the concentration of dissolved oxygen had fallen to 0.1 mg L^−1^ (Fig. 5b). From this point until the end of stratification Fe(II) was maintained at elevated levels.

Re-oxygenation of bottom waters occurred during winter in July 2016 (Fig. 2). The concentration of Fe decreased in all fractions (Fig. 5), consistent with the oxidation of Fe (II) species and the precipitation of Fe (III) hydroxides, most of which formed settleable gravitoids, and were removed from the water column. The measurements of dissolved Fe in July 2016 confirm that that while Fe was returned to the surface water by mixing, it was not DGT-labile (Fig. 5). Hence, the Fe data from July 2016 capture the suspension of Fe (III) colloids prior to aggregation and settling, a process which had continued to completion by October 2016.

Finally, the Fe(II) distribution (as determined by the Ferrozine method) was in broad agreement with the C_DGT_ data, but over-estimated Fe in all other fractions. It is important to note that Fe (III) can co-exist with Fe (II) at circumneutral pH values, and indeed thermodynamic disequilibrium between Fe(III)/Fe(II) species can be driven by microorganisms in some cases^42,43^. Although Viollier, *et al*.^47^. reported 3% higher absorption of Fe(II)-Ferrozine complex in their system, the results of the present study are broadly in line with the findings of Anastacio, *et al*.^48^ who overestimated Fe(II) as a result of photochemical reduction of Fe(III) complexes. To minimize the uncertainty in Fe(II) quantification here, the samples were pipetted to pre-prepared spectrophotometric cuvettes (containing Ferrozine) and treated for a short incubation time (<1 min), yet it was practically impossible to achieve the desired oxygen-free, dark conditions during measurements in an open boat. It is likely that the overestimation of Fe by the Ferrozine method was also due to interference by other divalent cations.

### Phosphorus depth distributions through time

**Figure 6:**
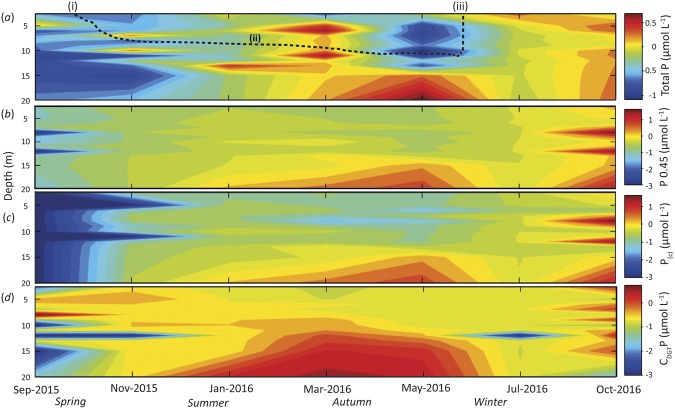
Time series of the depth-distribution of phosphorus (P) at log_10_ scale in operationally-defined water fractions from Lake Ngapouri, (**a**) Total P (μmol L^−1^), (**b**) 0.45 μm filtered fraction (dissolved P (μmol L^−1^)), (**c**) colloidal P_(c)_ (μmol L^−1^) and (**d**) C_DGT_ P (μmol L^−1^). Broken black line in (**a**) shows the timing of the onset (i) and end (iii) of stratified conditions in the lake as well as the approximate position of the thermocline (ii).

Mobile phosphorus has long been known to be intimately associated with particulate Fe in fluvial environments^20,49^ and interactions of the two have recently been shown for colloids in streams and aquifers^32^. During this study, the colloidal fraction of P (P_(c)_) was found to track the dissolved P concentration, demonstrating that P_(c)_ was dominant over dissolved species. Given that Fe in Lake Ngapouri was predominantly colloidal (Fig. 6), one line of evidence for the interaction of Fe and P in the water column would be the colloidal association of both elements. Indeed, an analysis of P size-distributions through time also confirms that DRP in this system (Fig. 6b) is mostly in the colloidal size range. Increases in P in both dissolved and colloidal fractions also coincided with increases in the DGT-labile P concentration (Fig. 6d).

DGT probes recorded three prominent instances of P release: discrete increases between 5–10 m depth during the onset of stratified conditions, and a sustained hypolimnetic increase in P through the summer period (Fig. 6c). It is notable that these increases in DGT-labile P were mirrored by high chlorophyll fluorescence and solvent-extracted chlorophyll *a* at the same or adjacent depths (Fig. 2).

### Coupling and decoupling of iron and phosphorus

Generally, under winter mixed conditions, the highly oxic water column was characterised by low concentrations of Fe (from being undetectable up to 11.6 μmol L^−1^ towards the bottom of the lake). Low dissolved Fe also coincided with low concentrations of *C*_DGT_ Fe (often close to zero) (Fig. 5). Thus, under mixed oxic conditions in the lake, the Fe data support the classical interpretation that Fe was present as colloidal Fe(III) hydroxides^50^ and was not DGT-labile because the Fe was held in the insoluble Fe(III) oxidation state.

During the summer-autumn stratified period the concentration of dissolved Fe (<0.45 μm) increased towards the bottom of the lake, reaching the highest value recorded in this study of 109 μmol L^−1^ at 20 m depth in May 2016, which coincided with a higher concentration of hypolimnetic P in the same fraction (7.51 μmol L^−1^). The same trend was observed in the P_(c)_ fraction, possibly indicating the release of adsorbed or co-precipitated P from Fe(III) hydroxides as a result of Fe(III) reduction and hydrolysis of Fe(II).

Phosphorus determined by inductively coupled plasma mass spectrometry (ICP-MS) from the filtered samples (<0.45 μm) was highest at 8 m depth under completely mixed conditions in October 2016 (74 μmol L^−1^). Notably, there was an increase in chlorophyll fluorescence at this depth during March 2016, which suggests that the higher value of P not associated with dissolution of Fe mineral phases, was instead driven by another process, such as the mineralisation of biomass.

**Figure 7:**
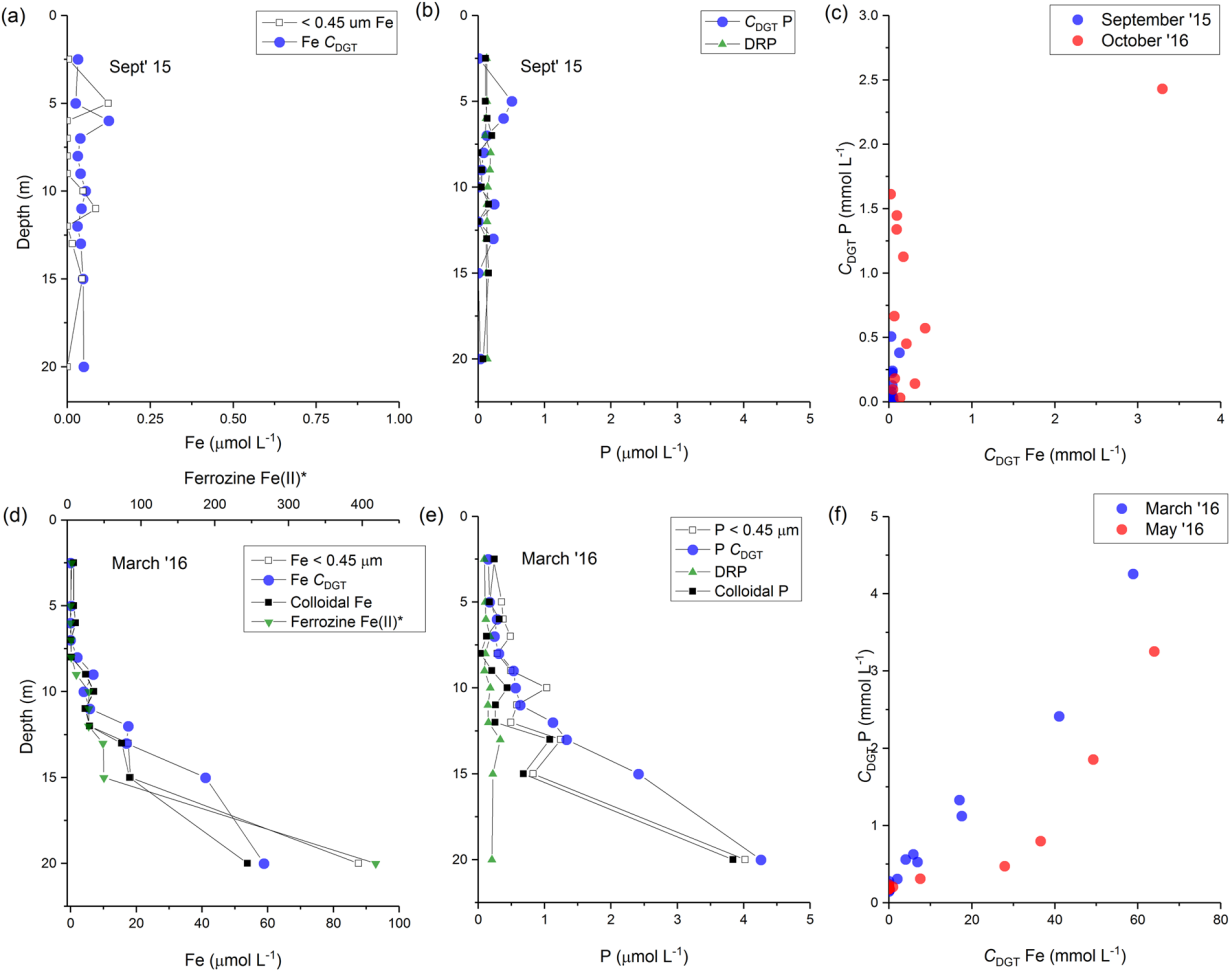
Depth distributions of iron (Fe), phosphorus (P) and cross-plots of DGT-labile Fe vs P in the winter mixed period (**a**–**c**) and summer-spring stratified period (**d**–**f**).

The coupling and decoupling of Fe and P is directly evident in the *C*_DGT_ data from the lake in the two seasons (Fig. 7). In the autumn (March-May), reductive dissolution of Fe colloids in the hypolimnion is implicated as the driver of elevated *C*_DGT_ Fe and P values. Dissolution of Fe colloids reached its zenith in March, and over the following two months the P: Fe ratio measured by DGT declined, probably consistent with biological uptake of P over that interval. Conversely, during the winter/spring mixed periods, phytoplankton blooms can be seen to have been entirely decoupled from Fe hydroxide reduction (Figs 2 and 5). The decoupling of P and Fe during the oxic intervals is consistent with the sequestration of P by insoluble Fe hydroxides, destabilisation of this colloidal suspension and sedimentation of the resulting Fe gravitoids. It appears that recently-sequestered Fe and P particles were likely suspended into the water column of the re-stratifying lake in the spring period (see colloidal distributions of P and Fe in the water column over these periods (Figs 5 and 6)), which could have been aided by bioturbation^51,52^.

At the onset of thermal stratification, the concentration of Fe^2+^ (inferred by the ferrozine assay) increased in the hypolimnion and gradually built up in summer through until March. In January 2015, Fe was predominantly colloidal in the hypolimnion, of which very little was DGT-labile. In addition, DGT-labile Fe was very closely related to Ferrozine Fe^2+^, implying that at this stage, Fe was present in a relatively inert form in the hypolimnion.

**Figure 8:**
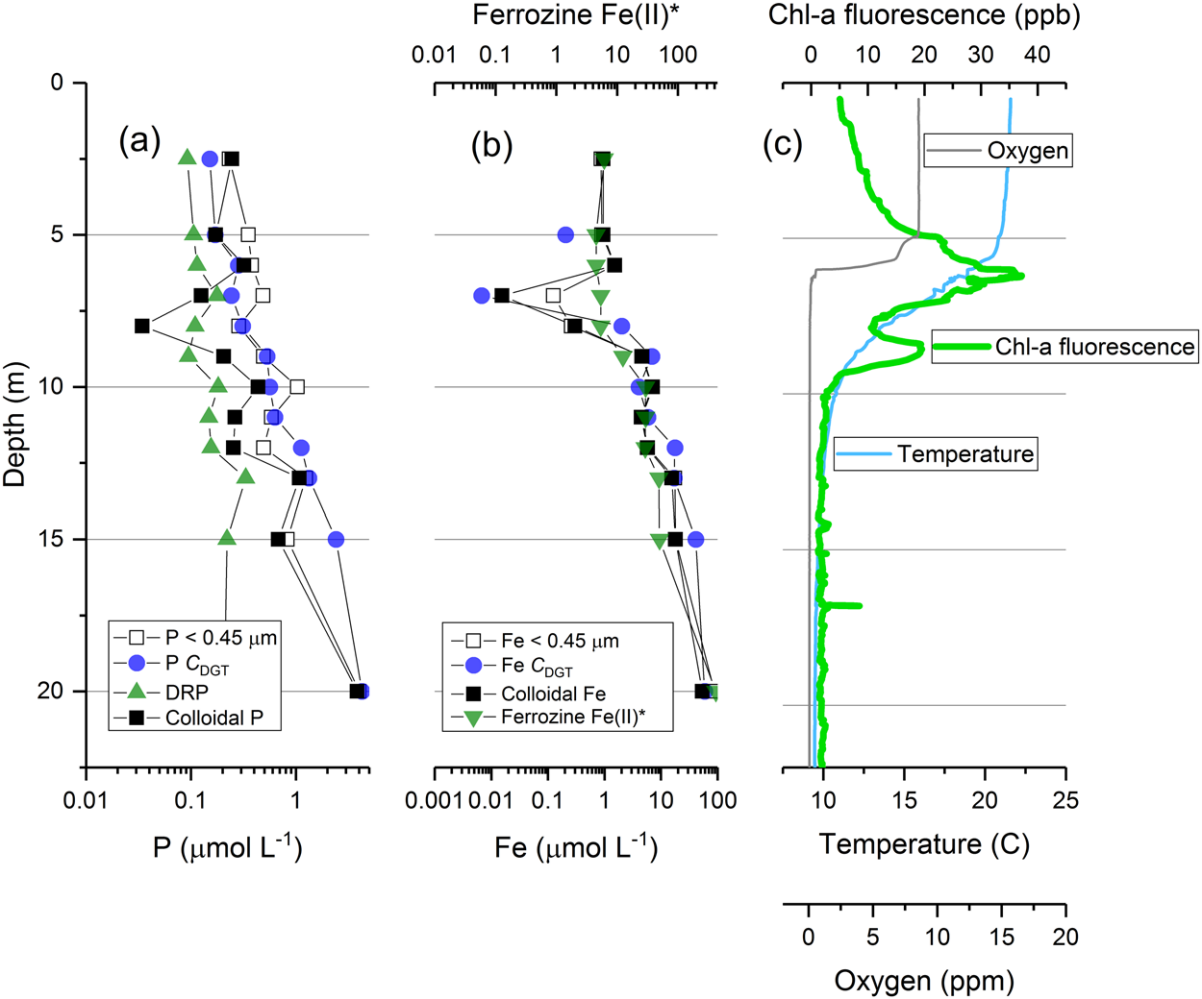
Depth distributions of (**a**) phosphorus and (**b**) iron fractions measured in Lake Ngapouri in March 2016. Panel (c) shows the corresponding CTD depth profiles for molecular oxygen, temperature and chlorophyll fluorescence.

During stratification in deep lakes, when the upper layer of the euphotic zone is nutrient-depleted and phytoplankton in the lower level are more likely to be limited by light supply, a so-called ‘deep chlorophyll maximum’ (DCM) develops–typically situated around the thermocline. This phytoplankton distribution has been linked to upward diffusion of nutrients from the enriched hypolimion waters^42,52,53^. In this study, when the stratification was still strongly maintained in March, the DCM was observed at 11 m in association with a strong depletion of dissolved Fe, colloidal Fe and DGT-labile Fe, as well as some depletion in colloidal P (Fig. 8) at the same stratum, likely reflecting biological uptake of this important micronutrient. Interestingly, the depletion of P was only observed in the colloidal P fraction, which would be consistent with targeted dissolution of Fe colloids to release Fe (an important micronutrient for phytoplankton). In the hypolimnion in March 2015, the DGT-labile fraction of Fe exceeded the concentration of Fe in the <0.45 μm fraction. This can be explained, but not demonstrated (due to a lack of particulate data), by the removal of Fe colloids as gravitoids^27^.

## Conclusion

This investigation suggests that colloidal Fe oxides are generated during mixing in monomictic lakes and possibly at the interface of oxic epilimnetic and hypolimnetic waters by cycles of microbial oxidation-reduction reactions. Our results also suggest that P was associated to these iron nanoparticles and that both Fe and P increased in concentration as reducing conditions deepened in the hypolimnion. These findings are in line with those of Gottselig, *et al*.^54^ and Jiang, *et al*.^55^ who demonstrated P associated with Fe oxide colloids in the size range of 1 to 20 nm in forest stream waters and agricultural soils, respectively, using coupled flow fractionation and ICP-MS, and Hartland *et al*.^32^ who showed dominant P adsorption to iron nanoparticles in streams and shallow (reduced) aquifers.

Further to this, we suggest that freshly precipitated Fe (oxy) hydroxides are small enough to diffuse through the APA hydrogel used in DGT solution probes at a rate comparable to that of solutes (at least when integrating over several days). These colloids bind P and thereby reduce the P concentration in the <100 KDa fraction. Based on elevated chlorophyll values at the same or adjacent depths, we conclude that colloidal P (and also Fe) is available for biological uptake and that DGT-labile P provides an analogous measure of P bioavailability. This interpretation is consistent with the findings of Montalvo, *et al*.^56^ who demonstrated that the colloidal P fraction was available for uptake by terrestrial plants. Our results confirm that iron nanoparticles play an important role in P transport and cycling in the lake systems, with important implications for phytoplankton community dynamics.

## Materials and Methods

### Site description

Lake Ngapouri is a small (0.19 km^2^) eutrophic lake located in Waikiti valley south of Rotorua, Bay of Plenty, New Zealand. It has a maximum depth of 24.5 m. A previous study reported increased concentrations of Fe, Mn, P and As in the anaerobic hypolimnion of the lake^44^. Being holomictic, monomictic and high in Fe, the lake was considered to be an ideal model system for examining P-Fe interactions.

### Field Methods

#### Water sampling

**Figure 9:**
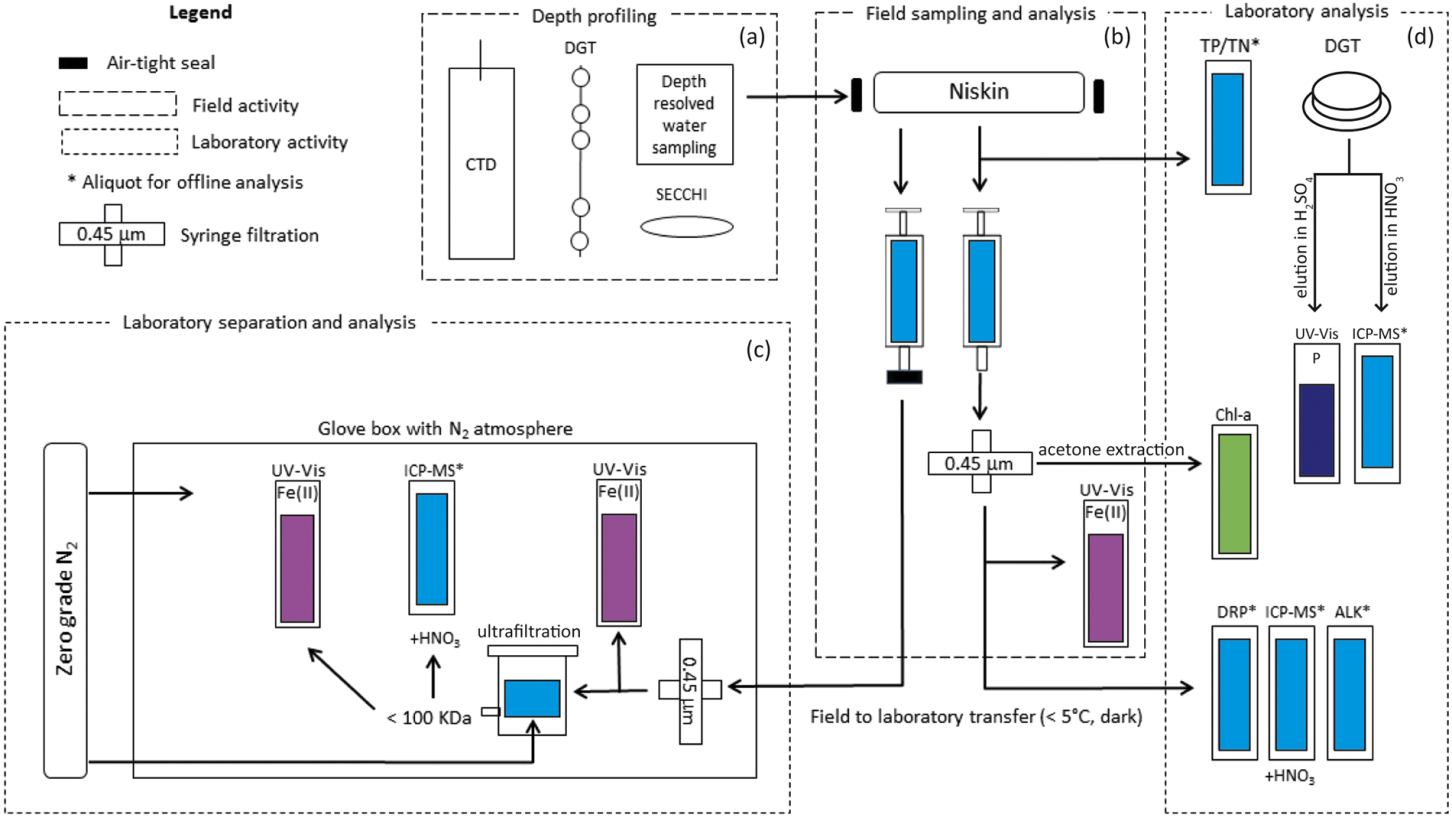
Water sampling and laboratory analysis workflow. Part (**a**) shows the range of in-field methods employed, (**b**) shows the water sample collection workflow including on-site filtration. Minimally-perturbed samples were collected in syringes and were capped for transport to the lab. Fe (II) was estimated on-site using Ferrozine analysis. Part (**c**) illustrates particle separation using stirred-cell ultrafiltration performed in a zero-grade N_2_ environment to avoid oxidative loss of Fe(II). Finally, part (**d**) shows the laboratory analysis procedure including the elution and subsequent analysis of DGT binding gels. *Denotes a water sample taken for off-line analysis.

Sampling of Lake Ngapouri took place every second month, from September 2015 to October 2016). Water samples were taken from 12 depths between 0 and 23 m, at the deepest point of the lake ~23 m depth). Figure 9 summarises the field-to-lab workflow.

A Van dorn water sampler (PVC Beta^TM^ Horizontal bottle, Envco, Auckland, New Zealand) was used to collect representative water samples from the discrete depth intervals. Vertical profiles of water column chemistry were constructed by sampling every meter (relative to the lake bottom) through the thermocline, as well as at two sampling points in the epilimnion and two in the hypolimnion. A conductivity, temperature and depth profiler (CTD; SBE 19 plus V2 SeaCAT profiler, Sea-Bird Electronics Inc., Washington) equipped with sensors for pH, dissolved oxygen, phtosynthetically active radiation (Licor Inc.) and chlorophyll fluorescence (Chelsea Instruments Ltd.) was used to determine the physiochemical character of the lake prior to and after each DGT deployment. Secchi depth was also measured to determine water clarity.

#### Filtration and preservation (in field)

Samples of 15 ml lake water were filtered on site by using 0.45μm syringe filters (Sartorius Stedim, Germany) in acid-washed bottles and were acidified on-site with ultrapure HNO_3_ to achieve 2% acidity in preparation for ICP-MS analysis (acid was prepared using low-temperature distillation in a Savillex (Eden Prairie, MN, USA) DST-1000 acid purification system). Overloading of filters was avoided as it can affect the pore size and may cause variable removal of particulates and colloids. To maintain the integrity of the collected water samples for major cation analysis, the screw-capped bottles were sealed using laboratory film and maximum care was taken to leave no head space in the bottles, to avoid oxidation. The bottles were then covered with aluminium foil and transferred in the dark at <5 °C to avoid photolysis. Additional samples were collected in pre-cleaned (acid/deionised water) syringes to determine the size distribution of the components of interest in the water column. Syringes were capped to avoid diffusion of O_2_ into the samples, and were then sealed with laboratory film and stored in the dark at or below 4 °C until analysis. As a measure of the extent of oxidative loss of Fe, the Ferrozine method (Hach, Colorado, USA) was used in the field to determine the dissolved Fe(II) concentration^47^. This analysis was conducted by taking an aliquot immediately from the Van dorn sampler which was directly transferred to a spectrophotometric cuvette already containing Ferrozine. This allowed immediate complex formation and thus minimised the potential for oxidative loss. The Ferrozine method was also used to measure the extent of post-sampling oxidation of the samples during handling in the laboratory and during separation of colloids by ultra-filtration.

Samples for Chl-*a* estimation were collected from each depth by filtering 50 mL of the water sample through 0.45 μm GF/C filters (Whatman, Maidstone,United Kingdom). The filters were then folded in half (sample side inward), individually wrapped in aluminium foil, kept in a container pre-wrapped with aluminium foil as extra precaution, and kept on ice until return to the laboratory, where the samples were frozen in dark and analysed using acetone extraction method within one week of collection.

### Diffusive gradients in thin films

To determine the concentration of labile trace elements and P in the lake water column, Chelex 100 (Bio Rad Laboratories Pty, Rosedale, Auckland, NZ) and ferrihydrite DGT devices were used in this study and prepared in-house after Zhang, *et al*.^45^ and Zhang and Davison^57^ respectively. The precision and accuracy of C_DGT_ values determined using DGT solution probes was confirmed before deployment in the lake and were found to be accurate to within 5% for Cd and P (Supplementary information, Tables S1 and S2). The assembled probes were transferred to airtight containers, immersed in 0.01 M NaNO_3_ and purged with zero grade N_2_ overnight to ensure removal of dissolved oxygen. This procedure was carried out in a pre-N_2_-purged glove box as an extra precaution. The dissolved oxygen level in the 1 M NaNO_3_ solution containing the DGT probes was below the level of detection of a DO probe (<1 mg L^−1^). The probes were then transferred to the field in airtight containers and the DO content was measured before deployment, and never exceeded 1 mg L^−1^.

Duplicate Chelex and ferrihydrite probes were strung onto a nylon rope at the required depth intervals, secured using cable ties, and immersed into the lake with minimum delay (Fig. S4). Probes were co-located with temperature loggers (UTBI-001, TidbiT _V_2 Temp Loggers (Onset Computer Corp., Cape Cod, MA, USA)) which, in addition to CTD data, were used to calculate the average temperature at each respective depth. During each deployment, a duplicate set of DGT probes which had not been purged with N_2_ was deployed to examine variations caused by oxygen trapped in the hydrogels (Fig. S6). The results showed no difference in concentration of analyte species bound to resin gels (Fig. S6).

After typically 4–5 days, the probes were retrieved from the lake water column, rinsed with DI water (resistivity 18.2 MΩ) and kept in sealed, pre-cleaned individual plastic bags which were transferred to the lab at <5 °C in the dark and kept under the same conditions until analysis.

### Laboratory Methods

The syringe samples were transferred to the N_2_ purged glove box for ultrafiltration in a stirred-cell ultrafiltration system (Amicon^R^, model 8400) equipped with Millipore Ultracel 100 KDa (molecular weight cut-off (MWCO)) regenerated cellulose ultrafiltration discs (Millipore Corporation, Billerica, MA, U.S.A.) which provide size exclusion above approximately 5 nm. Ultrafiltration for separation of colloids has been a focus of potential artefact^10,58^ and maximum care was taken to avoid these potential issues. The ultrafiltration unit was washed with 5% HNO_3_ and rinsed three times with deionized (DI) water before each use. Everything else, except for Chl-*a* estimation, was cleaned by soaking in 10% HCl overnight, rinsing with DI water, soaking in 10% HNO_3_ at least overnight and finally rinsing three times with DI water, and then air dried under a Class 100 laminar flow hood. Glassware was cleaned using Clean Aid and rinsed five times before Chl-*a* measurements. Prior to lake water filtration, the filter discs were washed with 50 mL of DI water and an aliquot was analysed as a blank for potential contamination from the filtration step. All blanks ranged between ±1 μg L^−1^ for P and ±1 μg L^−1^ for Fe.

Before ultrafiltration, the syringes were opened in the glove box and the Fe (II) content was again determined by complexation with Ferrozine^47^ to determine any Fe(II) loss during transportation and storage. The sampling trip was repeated when there was oxidative loss >3%. A 50 mL 0.45 μm filtered sample was then ultra-filtered under minimum back pressure (<30 kPa) of zero grade N_2_ and aliquots were collected in pre-cleaned (as described earlier) conical tubes that were kept open in the purging N_2_ glove box overnight to remove oxygen in the tubes. The filter membranes were exhaustively cleaned with 50 mL of DI water between every sample to avoid cross contamination and metal sorption to filters^59,60^. Aliquots were collected frequently to determine if any contamination had occurred. The filtration process was quick (5 ml/min) and the N_2_ flow rate was adjusted accordingly to avoid rupturing of the filter discs. The filtered fraction collected for major cation analysis was acidified in the glove box by using the same protocol as described under the “Filtration and preservation in the field” heading. The screw capped bottles were then analysed by ICP-MS calibrated using NIST-traceable certified reference materials from Inorganic Ventures (Christiansburg, Virginia).

Filter fractions were categorised on the basis of their relative pore size. However, because there is no direct relationship between molecular weight and the size of nanoparticles^60,61^ the size estimates based on filtration used in this study are approximate. The four fractions defined in this study are as follows: dissolved (corresponding to the sample filtered at 0.45 μm), colloidal (corresponding to the difference between 0.45 μm–100 KDa permeates), and nominally dissolved (corresponding to the 100 kDa permeate).

DGT probes were disassembled within 24 hrs of collection from the field and retrieved resins (binding gels) were immediately transferred to 1 M HNO_3_ in the case of Chelex and 0.25 M H_2_SO_4_ for ferrihydrite. The ferrihydrite eluent was analysed using the molybdenum blue method^62^. Eluents of Chelex gels were analysed by ICP-MS.

#### Characterization of colloids

Colloids from Lake Ngapouri were characterized by using transmission electron microscopy (TEM) and atomic force microscopy (AFM). TEM samples were prepared by placing ~30 μl of water sample on support films (Formvar/carbon coated 200-mesh copper, obtained from ProSciTech, QLD, Australia) and allowed to dry under a zero-grade N_2_ atmosphere. Freshly cleaved mica sheets (ProSciTech, QLD, Australia) were placed in 15 mL of water sample for 24 hr immediately after return to the laboratory and then allowed to dry under the same N_2_ atmosphere in screw-capped bottles (thereby avoiding deposition of settling particles). We recognize the possible salt crystallization on the surface of mica sheets during sample preparation steps^61^ and so the results are interpreted accordingly.

#### Instrumental method

Both filter fractions, i.e. dissolved (<0.45 μm) and nominally dissolved (<100 KDa) were analysed on a Perkin Elmer (Waltham MA) quadrupole ICP-MS calibrated using certified reference materials. Internal standards of known concentration were also analysed to determine instrumental drift during analysis. Blanks (DI water, 18.2 MΩ) and procedural blanks (DI water transported to the field with samples) were also analysed following every sampling campaign and reported values of most trace elements were very low. The average concentration of Fe was 0.168 μmol L^−1^ for the nominally dissolved fraction i.e. <100 KDa and 0.179 μmol L ^−1^ for colloidal fraction, i.e. 0.45 μm–100 KDa permeates.

#### Flow injection analyser

Dissolved reactive phosphorus (from 0.45 μm) was analysed using standard colorimetric methods (APHA, 1998)^63^ on a Lachat QuickChem flow injection analyser (Zellweger Analytics Inc.). A range of standards was prepared in DI water to confirm the analytical detection limit, which was found to be 0.004 mg L^−1^. DI water was used as a blank. The total phosphorus concentration was determined in unfiltered lake water sample by using the same method as described above but after persulphate digestion^62^.

#### Atomic Force Microscopy

AFM imaging was carried out by using an XE-100 atomic force microscope (Park systems Corporation, Suwon, Korea). The analyses were carried out in a true noncontact mode under ambient conditions using silicon cantilevers with a spring constant of 42 N m^−1^ (PPP-NCHR, Park Systems Corp.) and images were recorded in topography mode. Images were collected from 15 to 20 arbitrary areas on the film and were recorded in 256 × 256 pixel resolution. Particles were then grouped at 5 nm intervals based on measured heights.

#### Fluorometric estimation of Chl-a

The frozen glass fibre filter was transferred to clean tubes in very dim light and Chl*-a* was extracted by adding 5 mL buffered acetone. The filter was then ground by using an IKA® T10 basic Ultra-Turrax homogenizer (Thermo Fisher Scientific, Auckland, New Zealand). Five mL of buffered acetone was then added to wash any left overs off the grinding tip. The samples were allowed to steep for ~24 hr, shaking at least once during this time. After the steeping period, the samples were shaken and centrifuged for 10 min at 3300 rpm, then allowed to stand for ~30 mins in the dark at room temperature before reading.

Before proceeding to measure Chl-a, glass cuvettes were visually checked for any scratches. 5 mL of buffered acetone was read as a blank, followed by addition of 150 μL of 0.1 N HCl. Five mL of the sample was decanted off and read at the “High” setting, then diluted with buffered acetone when the reading was “Over”, with the dilution factor was subsequently taken into account in calculations. 150 μL of 0.1 N HCl was then added, mixed by gently tapping the cuvette, and re-read after 90 seconds.

### Calculations

The concentration of colloidal Fe and Colloidal P was calculated according to Equations 1 and 2 respectively:

1 $${\mathrm{Fe}}_{(c)}={{\rm{Fe}}}_{({d})}-{{\rm{Fe}}}_{(100{\rm{KDa}})}$$

2 $${{\rm{P}}}_{(c)}={{\rm{P}}}_{(d)}-{{\rm{P}}}_{(100{\rm{KDa}})}$$

where Fe_(c)_ represents the colloidal fraction and Fe_(d)_ is filtrate through 0.45 μm filter membrane and Fe_(100KDa)_ is the permeate filtering through 100 KDa filter membranes.

#### Calculating C_DGT_

C_DGT_ concentrations were calculated for each resin disc by the following steps:

First, the mass (M) of P and Fe was calculated in respective resin gels using Equation 3

3 $${\rm{M}}={\rm{Ce}}({{\rm{V}}}_{{\rm{acid}}}+{{\rm{V}}}_{{\rm{gel}}})/fe$$

where *Ce* is concentration of P or Fe in resin gel eluent, *V*_*acid*_ is the volume of acid added to the resin gels, *V*_*gel*_ is volume of the resin gel and *fe* is the elution factor of P or Fe.

The DGT concentration was then calculated by Equation (4)

4 $${{\rm{C}}}_{DGT}=M\Delta g/(DtA)$$

where *Δ*g is diffusive boundary layer (thickness of diffusive gel + thickness of the filter membrane), D = diffusion coefficient of the elements in gel as reported by Zhang and Davison^37^, t is deployment time (s) and A is exposure area (cm^2^). This equation was used for calculating C_DGT_ value in the solution probes by using geometric area of standard solution DGT holders. When DGTs are immersed in water, a thin layer of water which is in line with the exposed window of the device is usually inactive and transport of material through this layer, known as Diffusive Boundary Layer (DBL), is believed to happen through diffusion only. The effect of DBL was minimised during this study by deploying devices for longer periods of time. Moreover, a 0.8 mm thick layer of diffusive gel along with 0.14 mm thick filter membrane was used for simplicity.

#### Calculating Chl-*a*

The concentration of Chl-a was calculated by using the following equation:

$$Chl-a({{\rm{\mu }}\mathrm{gL}}^{-1})={{\rm{F}}}_{{\rm{s}}}[({\rm{r}}/({\rm{r}}-1))\,({{\rm{R}}}_{1}-{{\rm{R}}}_{2})]\,[({{\rm{V}}}_{{\rm{e}}}{\rm{Xdf}})/{{\rm{V}}}_{{\rm{f}}}]$$

where F_s_ is response factor of the fluorometer, R_1_ and R_2_ are readings before and after the acidification step, respectively, r is the acidification coefficient, d_f_ is dilution factor, V_e_ is extraction volume and V_f_ is the volume of water filtered.

The depth of the thermocline was determined by calculation of dT/dz where z is the depth of water column increasing downwards^42^ and T is temperature. The water column was considered fully mixed when dT/dz was > −0.225 °C m^−1^ Data from CTD profiles were averaged over 0.125 m intervals to minimize noise. The top 5 m of the water profile was not included in thermocline calculations to avoid temporary diurnal thermoclines formed under calm conditions during daytime.

## Electronic supplementary material

Supplementary Information
