# Supplementary material for: Regulation of phosphorus bioavailability by iron nanoparticles in a monomictic lake
Source: Sci Rep. 2018 Dec 10;8:17736. doi: 10.1038/s41598-018-36103-x (PMC6288117; doi:10.1038/s41598-018-36103-x)
Supplement: Supplementary file 1 — Supplementary Information [file 41598_2018_36103_MOESM1_ESM.docx]

**Supplementary information**

**Regulation of phosphorus bioavailability by iron nanoparticles in a monomictic lake**

Saeed, H^1^; Hartland, A^1*^; Lehto, N^2^; Baalousha, M^3^; Sikder, M^3^; Sandwell, D^1^; Mucalo, M^4^; Hamilton, DP^5^.

**Addresses**

^1^Environmental Research institute, School of Science, Faculty of Science and Engineering, University of Waikato, New Zealand

^2^Dept. of Soil and Physical Sciences, Faculty of Agriculture and Life Sciences, Lincoln University, New Zealand

^3^Center for Environmental Nanoscience and Risk, Department of Environmental Health Sciences, University of South Carolina, Columbia, SC, United States

^4^Chemistry, Faculty of Science and Engineering, University of Waikato, Hamilton, New Zealand

^5^Australian Rivers Institute, Griffith University, Australia

*Corresponding author: [adam.hartland@waikato.ac.nz](mailto:adam.hartland@waikato.ac.nz)

**Summary**

**Figure S1.** Depth profiles of physicochemical characteristics, different fractions of P and Fe of lake Ngapouri from September 2015 to October 2016.

**Figure S2.** pH of the water column of Lake Ngapouri from September 2015 to October 2016.

**Figure S3.** Temperature profile of Lake Ngapouri based on temperature loggers programmed to collect data every 30 minutes.

**Figure S4.** DGT deployment in the field, probes with black precipitates are from near the bottom of the lake and show the accumulation of metal sulphides.

**Figure S5** Standard curve for the estimation of HPO_4_^2-^ by the molybdenum blue method.

**Table S1** Test deployment of ferrihydrite DGT probes in 200ppb HPO_4_^2-^ solution for known time at room temperature

**Table S2** Test deployment of chelex DGT probes in 200 ppb HPO_4_^2-^ solution for known time at room temperature.

**Figure S6.** Results of Chelex DGT paired measurements during summer stratification.

**Fig S7.** Comparison of Fe (II) measurements by the Ferrozine method, “Fe”, “Fe_b” and “Fe _c” represents “in field measurements”, “after bringing to the lab ***but*** before ultrafiltration” and “after ultrafiltration through 100KDa in the glove box” respectively.

**
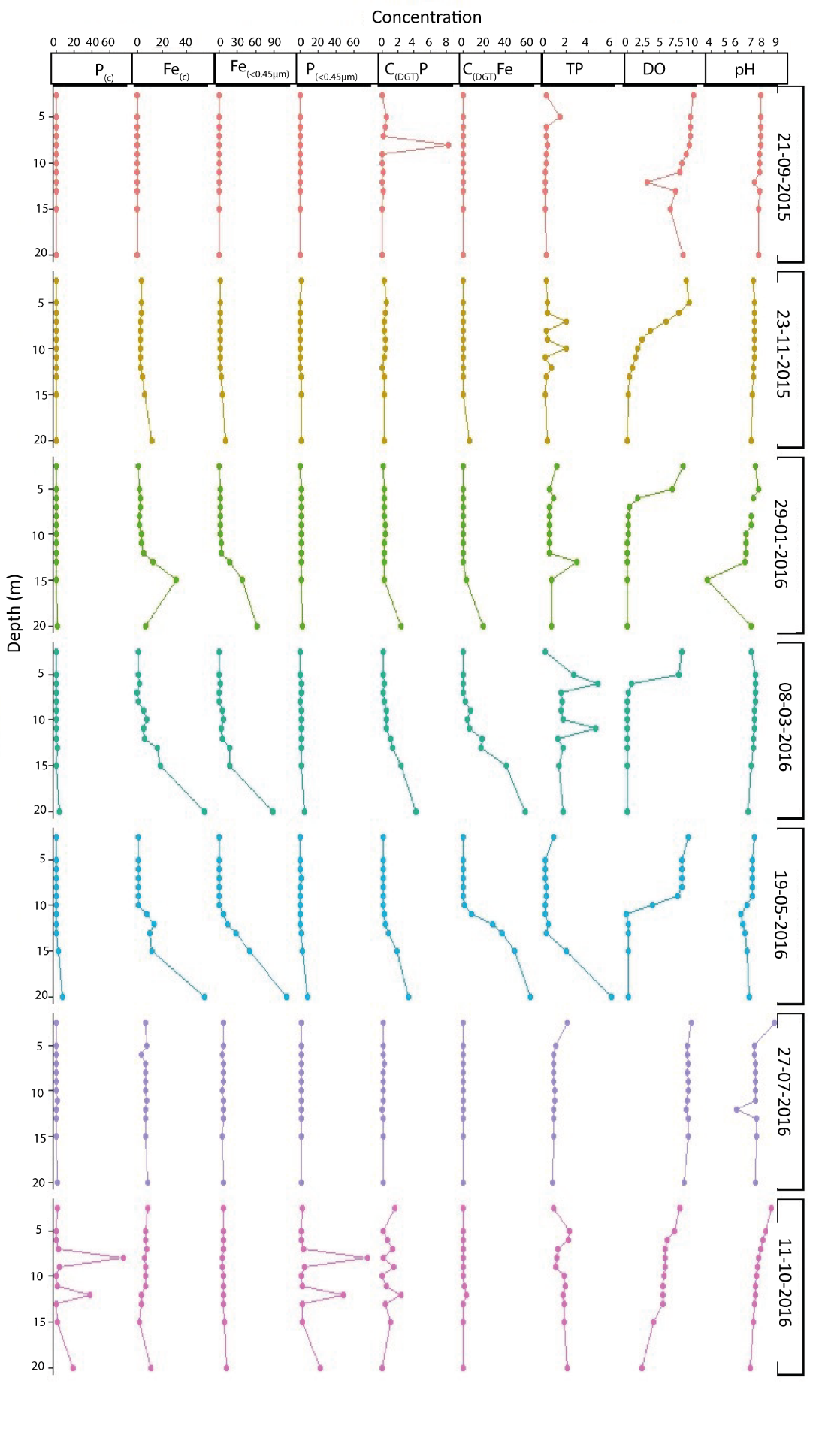
**

**Figure S1**. Depth profiles of physicochemical characteristics and different fractions of P and Fe in Lake Ngapouri from September 2015 to October 2016. The concentration values are in μM L^-1^ and depths are in meters.


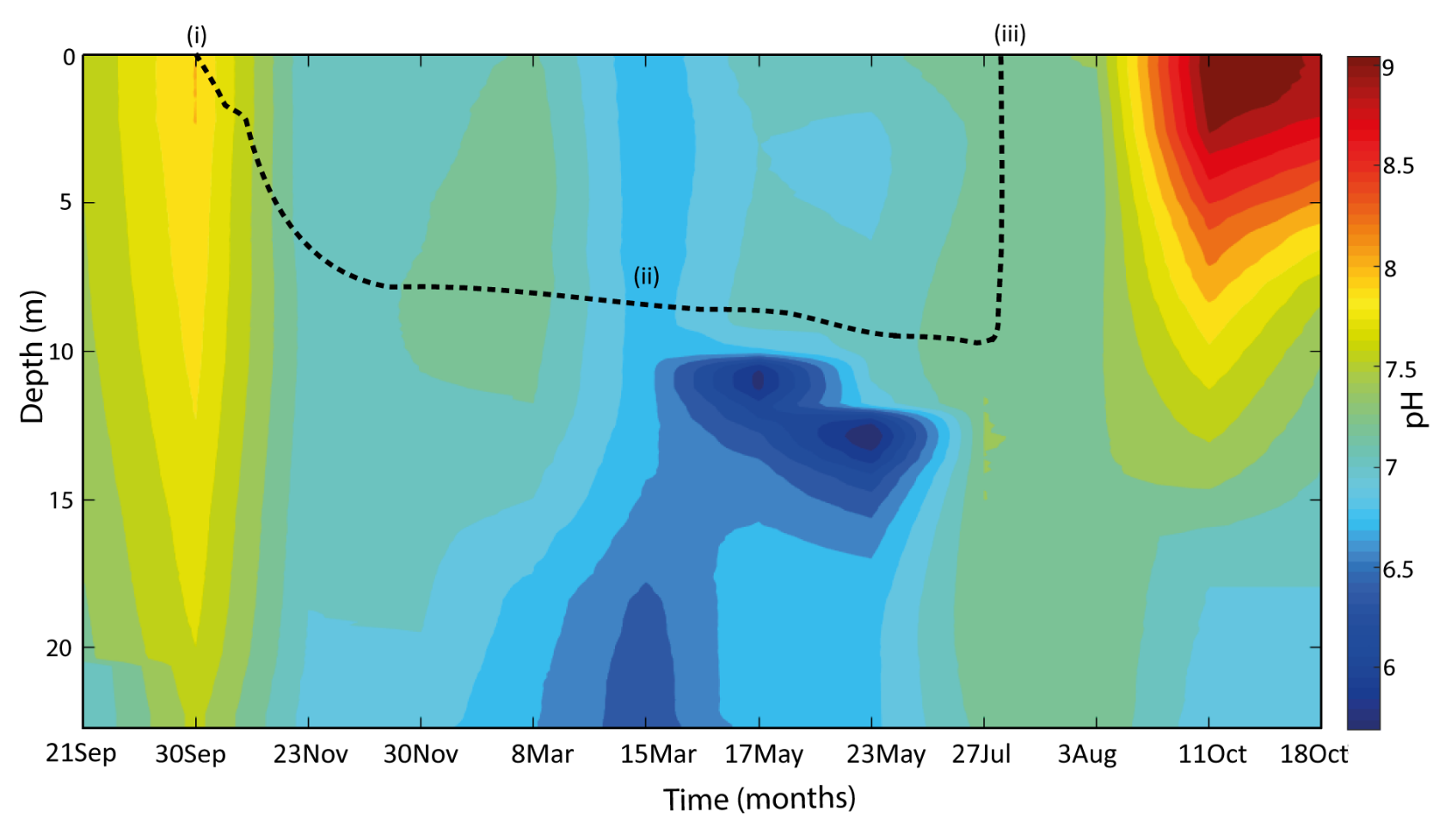


**Figure S2.** pH of the water column of Lake Ngapouri between September 2015 and October 2016. Data are missing from January 2016 due to a faulty probe. Broken black lines show the timing of the onset (i) and end (iii) of stratified conditions in the lake as well as the approximate position of the thermocline (ii). Data from January 2016 are not included in the plot due to a probe malfunction in this month.

**
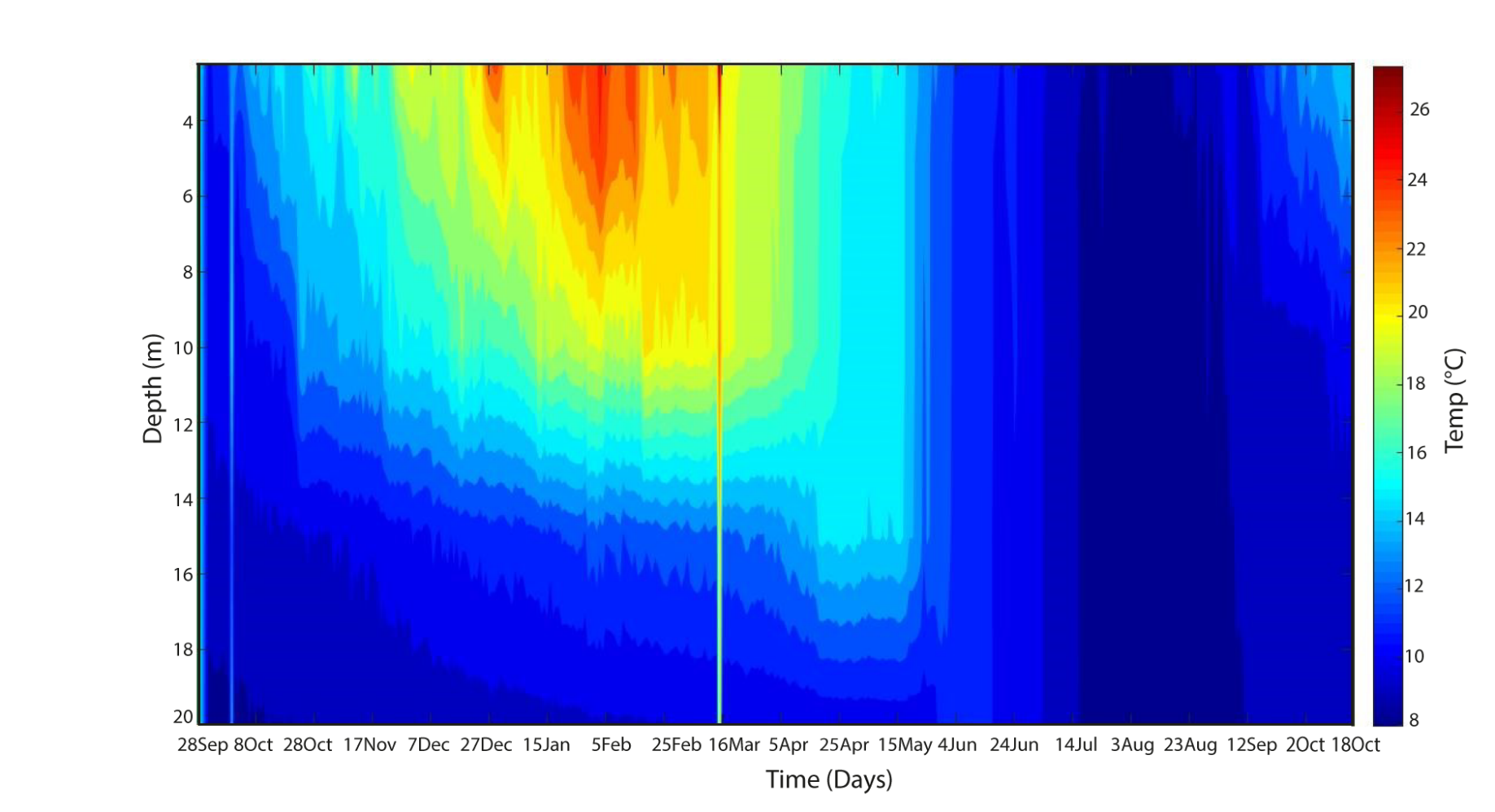
**

**Figure S3.** Temperature profile of Lake Ngapouri between September 2015 and October 2016 recorded using Hobo Tidbit temperature loggers programmed to collect data every 30 minutes.


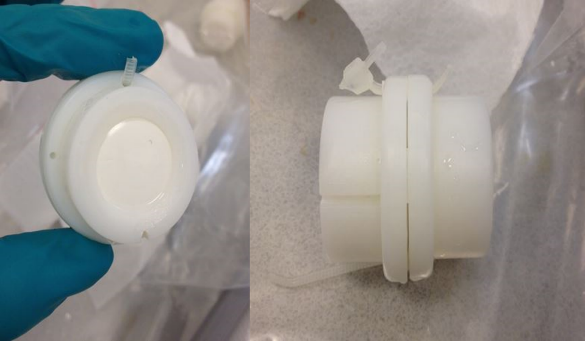

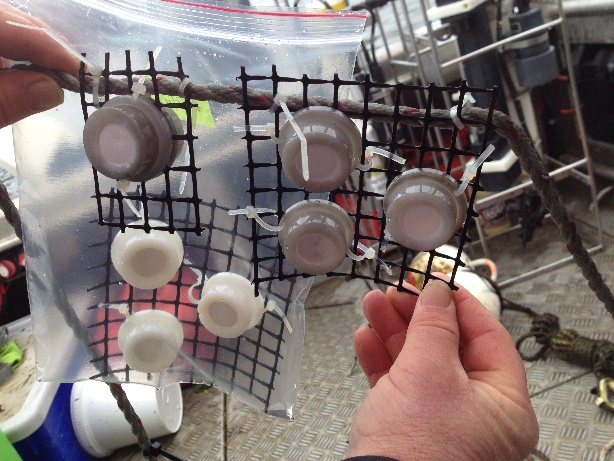


**Figure S4**. DGT deployment in the field. DGT probes coated with dark precipitate are from close to the benthic nepheloid layer and provide evidence for the formation of metal sulphides at this depth.

**Test deployment of DGT probes in a standard solution**

The gel solution was prepared by mixing 15% of acrylamide and 0.3% cross linker V/V (from DGT research ltd, Lancaster). A 10% ammonium persulphate (W/V), from now on referred to as “the initiator”, was prepared fresh by dissolving 0.1 g salt to 1 mL of deionised (DI) water. 70 μL of initiator was added to 10 mL of gel solution followed by 20 μL of TEMED catalyst and was immediately cast between two glass plates separated by sheet styrene spacers and allowed to polymerise at about ~42-44^O^C.

The ferrihydrite solution probes were prepared after Zhang *et al* ^(1)^. Briefly, to prepare the ferrihydrite slurry, 0.1 M Fe^3+^ was titrated with 1M NaOH while stirring vigorously until the pH reached to ~7 (never allowed to exceed 7). Ferrihydrite was allowed to settle and the surface water was removed carefully by pipetting. The ferrihydrite slurry was then washed at least three times with deionised water and was stored in dark at 4^o^C until used. 2 g of this slurry was added to 10 mL of gel solution following the same casting procedure as described earlier.

To prepare chelex gel sheets, 4 g of hydrated Chelex 100 resin in the Na form (Bio-Rad, Australia) was added to 10 mL of gel solution followed by 60 μL of initiator and 15 μL TEMED, respectively, and cast and polymerised as described in previous paragraphs in this document. All gels were hydrated in deionised water for 24 hrs before use to obtain stable dimensions and remove unreacted reactants or impurities.

To test the precision and accuracy of the ferrihydrite piston probes the assembled probes were immersed in 200 ppb P solution (from here on referred to as the “immersion solution”) prepared by using KH_2_PO_4_ (Merck, Darmstadt, Germany). 3 L of immersion solution was prepared fresh before each deployment and vigorously stirred for at least 90 minutes before immersion of the probes. The DGT probes placed in a holder and placed in the immersion solution and the stirring speed was adjusted to avoid the formation of a vortex. A 10 ml aliquot of immersion solution was collected at the start and end of the test was analysed by the molybdenum blue method for PO_4_^2-^ concentration.

**Figure S5.** Standard curve for the estimation of HPO_4_^2-^ by the molybdenum blue method.

| Sample ID | Absorbance | Conc.(ppb) | C_DGT_ P(ppb) | t _(sec)_ | Temp (^o^C) | Diffusion coefficient |
| --- | --- | --- | --- | --- | --- | --- |
| blank 1 | 0.008 | 0 |  |  |  |  |
| blank 2 | 0.01 | 0 |  |  |  |  |
| blank 3 | 0.009 | 0 |  |  |  |  |
| Probe 1 | 0.095 | 132.57 | 180.59 | 58500 | 22 | 5.57E-06 |
| Probe 2 | 0.087 | 121.14 | 170.62 |  |  |  |
| Probe 3 | 0.096 | 134.00 | 154.65 |  |  |  |
| Probe 4 | 0.092 | 126.86 | 162.63 |  |  |  |
| Probe 5 | 0.092 | 126.86 | 162.63 |  |  |  |
| Sol. _(i)_ | 0.124 | 174 |  |  |  |  |
| Sol. _(f)_ | 0.117 | 164 |  |  |  |  |

**Table S1**. Test deployment of DGT piston probes in 200 ppb HPO_4_^2-^ solution for a known time at room temperature (22^O^C). Sol_(i)_ and Sol_(f)_ represent the initial and final concentrations of P in the deployment solution, respectively. Conc. Is the concentration of P recovered in ferrihydrite elution solution. The initial and final pH of the immersion solution was 6.9 and 6.8 repsectively.

Chelex DGT probes for trace metal uptake were tested by immersing five probes in a 200 ppb Cd solution prepared from CdCl_2_. Probes were deployed for a known time and the temperature was recorded at the beginning and end of the experiment. Aliquots of the initial and final immersion solution was acidified and alaysed by ICP-MS. Chelex gels were eluted in 1 M HNO_3_ overnight at room temperature and analysed by ICP-MS.

| Sample ID | Conc.(ppb) | C_DGT_Cd | t _(sec)_ |
| --- | --- | --- | --- |
| blank 1 | 0.22 |  |  |
| blank 2 | 0.21 |  |  |
| blank 3 | 0.23 |  |  |
| Probe 1 | 23.13 | 236.71 | 21300 |
| Probe 2 | 23.89 | 244.56 |  |
| Probe 3 | 24.79 | 253.86 |  |
| Probe 4 | 23.83 | 243.94 |  |
| Probe 5 | 24.56 | 251.49 |  |
| Sol. _(i)_ | 260.34 |  |  |
| Sol. _(f)_ | 214.04 |  |  |

**Table S2**. Test deployment of Chelex DGT probes in 200 ppb Cd solution for known time at 22^o^C. Sol_(i)_ and Sol_(f)_ represent the initial and final concentrations of Cd in the immersion solution. The immersion solution was stirred for at least 90 minutes before deployment.


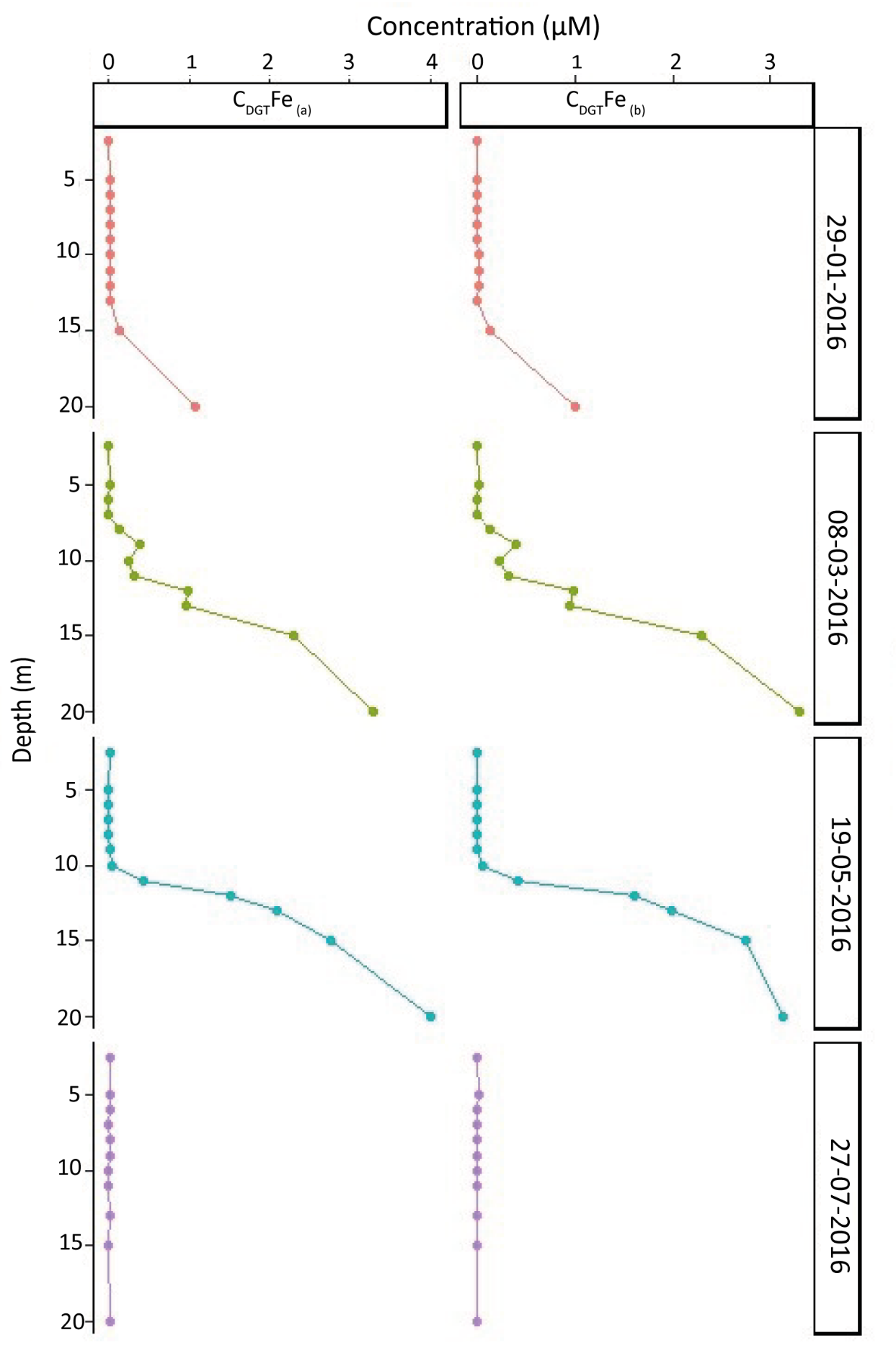


**Figure S6**. Results of C_DGT_ Fe values (μM). The probes were deployed in pairs and C_DGT_Fe_(b)_represents the results from the probes purged with zero grade N_2_ overnight before deployment. These results highlight the remarkable reproducibility of fine-scale structure in the metal depth profiles measured by DGT.


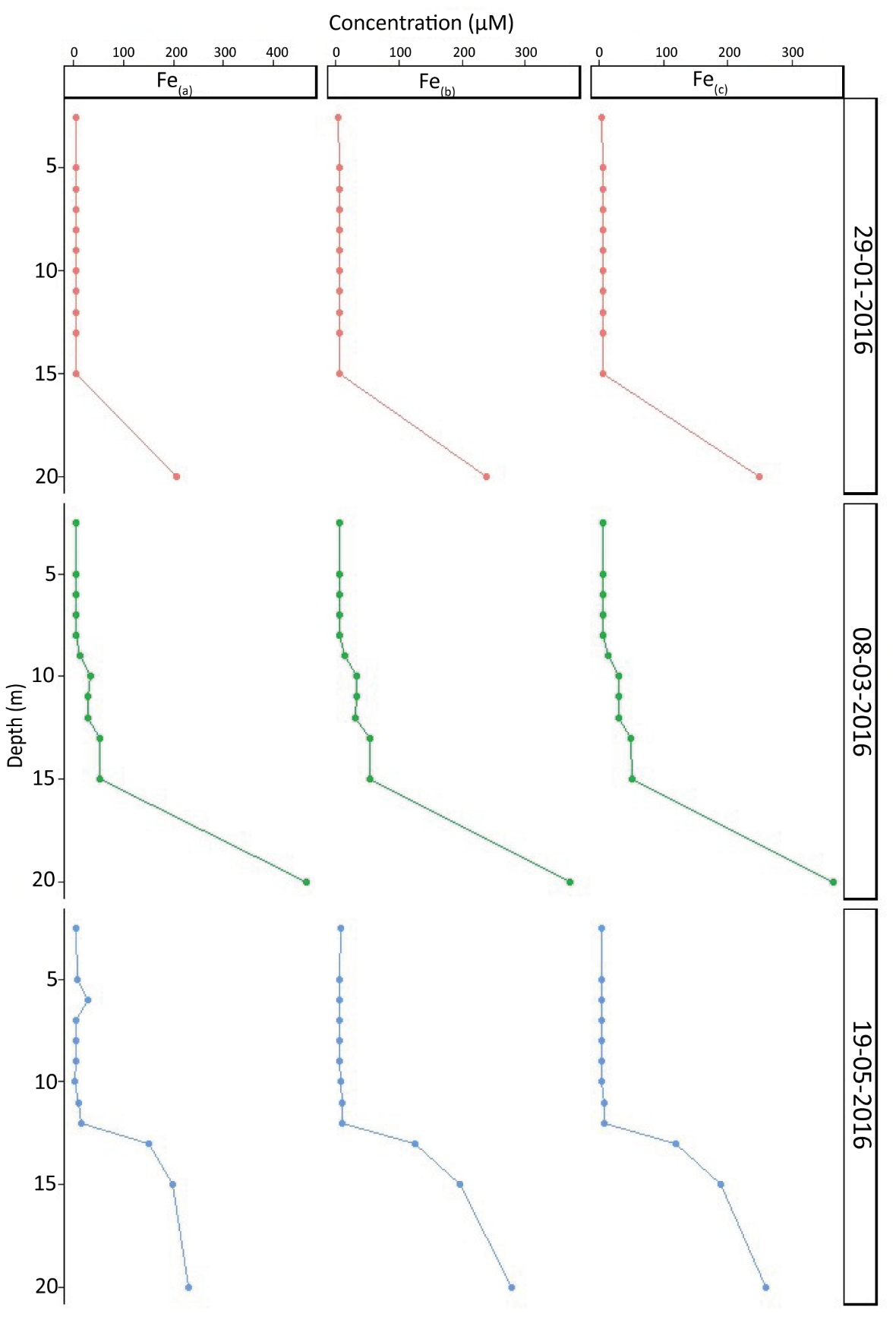


**Figure S7.** Comparison of Fe(II) concentrations (μM) determined by the Ferrozine method, “Fe _(a)_”, “Fe_(b)_” and “Fe_(c)_” represent “in field measurements”, “after bringing to the lab ***but*** before ultrafiltration” and “after ultrafiltration through 100 KDa in the glove box”, respectively.

**References**

1. Zhang H, Davison W, Gadi R, Kobayashi T. In situ measurement of dissolved phosphorus in natural waters using DGT. Analytica Chimica Acta. 1998;370(1):29-38.

2. Zhang H, Davison W. Diffusional characteristics of hydrogels used in DGT and DET techniques. Analytica Chimica Acta. 1999;398(2):329-40.
